# Novel 6-Month Treatment for Drug-Resistant Tuberculosis, United States 

**DOI:** 10.3201/eid2701.203766

**Published:** 2021-01

**Authors:** Connie A. Haley, Patricia Macias, Supriya Jasuja, Betsy A. Jones, Marie-Claire Rowlinson, Roshni Jaimon, Pennelyn Onderko, Elaine Darnall, Maria E. Gomez, Charles Peloquin, David Ashkin, Neela D. Goswami

**Affiliations:** University of Florida, Gainesville, Florida, USA (C.A. Haley, M.E. Gomez, D. Ashkin);; Cook County Department of Public Health, Chicago, Illinois, USA (P. Macias, S. Jasuja, R. Jaimon, P. Onderko);; Florida Department of Health, Jacksonville, Florida, USA (B.A. Jones, M.-C. Rowlinson, D. Ashkin);; Illinois Department of Public Health, Springfield, Illinois, USA (E. Darnall);; University of Florida College of Pharmacy, Gainesville (C. Peloquin);; Centers for Disease Control and Prevention, Atlanta, Georgia, USA (N.D. Goswami)

**Keywords:** tuberculosis and other mycobacteria, antimicrobial resistance, drug monitoring, bedaquiline, pretomanid, linezolid, BPaL, Nix-TB, United States, bacteria

## Abstract

The US Food and Drug Administration approved a 6-month regimen of pretomanid, bedaquiline, and linezolid for extensively drug-resistant or multidrug-intolerant tuberculosis after a trial in South Africa demonstrated 90% effectiveness 6 months posttreatment. We report on a patient who completed the regimen using a lower linezolid dose.

A woman from eastern Europe in her late 40s undergoing treatment for stage III cervical adenocarcinoma was found to have a right upper lobe pulmonary nodule. Pathology from tissue biopsy demonstrated necrotizing granulomas and numerous acid-fast bacilli (AFB); the sample was PCR positive for *Mycobacterium tuberculosis*. Adjuvant chemotherapy was held, and the patient was referred to the local public health department. The patient provided informed written consent for publication of her case study.

On evaluation, the patient was asymptomatic without physical findings and reported no previous diagnosis or treatment of tuberculosis (TB) disease or latent TB infection. Results of serologic testing for HIV and viral hepatitis B and C were negative. She had a mild chronic anemia and transient transaminitis during chemotherapy (peak alanine aminotransferase 215 IU/L; aspartate aminotransferase 185 IU/L). Three sputum samples were negative by AFB smear and culture; 1 was tested by PCR and was *M. tuberculosis* negative.

Treatment with rifampin, isoniazid, pyrazinamide, ethambutol and pyridoxine was initiated. Lung biopsy cultures grew *M. tuberculosis,* and GeneXpert MTB/RIF assay (Cepheid, https://www.cepheid.com) detected an *rpoB* mutation indicating likely rifampin resistance. Rapid molecular detection of drug resistance and growth-based drug susceptibility testing performed by the Centers for Disease Control and Prevention (CDC) and Florida Bureau of Public Health Laboratories yielded concordant results. We detected resistance to isoniazid, rifampin, the fluoroquinolones (levofloxacin and moxifloxacin), and an injectable (kanamycin), confirming a diagnosis of extensively drug-resistant TB (XDR TB). Resistance was also detected for pyrazinamide but not for ethambutol, bedaquiline, or linezolid. The patient and her medical providers, in consultation with a CDC-funded TB Center of Excellence (COE, https://www.cdc.gov/tb/education/tb_coe), determined that her best treatment option was a 6-month all-oral regimen of bedaquiline, pretomanid, and linezolid (BPaL).

BPaL was approved by the US Food and Drug Administration (FDA) on August 14, 2019, based in part on results from the Nix-TB trial in South Africa, which included patients with XDR TB or multidrug-resistant (MDR) TB who failed or were intolerant of prior therapy (*1*). Pretomanid, the novel agent in the regimen, is a nitroimidazooxazine that blocks cell-wall production in actively replicating MTB organisms and acts as a respiratory poison and protein synthesis inhibitor to kill nonreplicating persister organisms (*2*). Bedaquiline is a diarylquinoline that inhibits mycobacterial adenosine triphosphate synthase in replicating and persister organisms, and linezolid is an oxazolidinone that also inhibits protein synthesis (*3**,**4*). The combined activity of BPaL enables cure in a far shorter period compared with currently recommended 18- to 24-month MDR TB regimens (*5*). In the Nix-TB trial, BPaL produced favorable outcomes in 98/109 (90%) patients at 6 months posttreatment (*1*); in addition, little preexisting resistance to bedaquiline, pretomanid, or linezolid has been reported (*4**,**6*).

Because pretomanid was not yet commercially available in the United States, the TB Alliance required an FDA-approved single-patient investigational new drug application and provided 6 months of pretomanid acquired internationally. A bridging regimen of bedaquiline, linezolid, moxifloxacin, cycloserine, clofazimine, and ethambutol was initiated for 2 weeks, then was narrowed to BPaL when pretomanid arrived.

For this patient, we initiated linezolid at 600 mg/d, given the toxicity of the Nix-TB dose of 1,200 mg/d, the patient’s paucibacillary disease, and TB COE’s experience with linezolid dosing (*1**,**4**,**7**,**8*). Therapeutic drug monitoring performed at the University of Florida Infectious Diseases Pharmacokinetic Laboratory (https://idpl.pharmacy.ufl.edu) was used to maintain a linezolid peak of 12–26 µg/mL and trough <2 µg/mL to reduce drug-induced toxicity (*4**,**9*).

The patient received outpatient BPaL treatment 7 days a week by directly observed therapy. We assessed liver, renal, hematologic, and neurologic function plus QTc intervals at baseline and every 2–4 weeks during treatment (Table 1). A few weeks into therapy, the patient’s linezolid level 18 hours postdose was measured at 7.62 µg/mL (serum trough level at 24 hours was likely lower but was still higher than expected) (Table 2). To reduce the trough while maintaining a peak serum level 4–16 times over her *M. tuberculosis* isolate’s linezolid MIC of 0.12 μg/mL, we extended the linezolid dosing interval to 600 mg every Monday, Wednesday, and Friday. A subsequent linezolid trough at 48 hours was calculated at <2 µg/mL. The patient completed 182 doses of BPaL over 26 weeks without treatment interruptions. Other than mild nausea that responded to pantoprazole, she had no adverse events or notable changes in laboratory values or electrocardiographs. Nine months after completion, the patient remained well; the state health department expects to closely monitor her for recurrent TB for 24 months after BPaL completion.

**Table 1:** Molecular susceptibility sequencing results and therapeutic drug monitoring data from treatment of XDR TB, Florida, USA*

Drug (dose)	Sequencing result	Date drug level drawn	Trough, µg/mL	2h postdose level, µg/mL	6h postdose level, µg/mL	Typical peak serum concentration, µg/mL
Bedaquiline (200 mg MWF)	No *atpE* (ORF) mutation detected; no Rv0678/mmpR (ORF) mutation detected	2019 Nov 13	0.51 (42.25 h postdose)	1.40	1.42	1.2–1.8 (5–6 h postdose, maintenance phase)
N-monodesmethyl bedaquiline (metabolite)	NT	2019 Nov 13	0.22 (42.25 h postdose)	0.24	0.27	NT
Pretomanid (200 mg/d)	NT	2019 Nov 13	2.07 (18.25 h postdose)	3.43	2.98	2.3–4.3 (5–6 h postdose, at steady state)
Linezolid (600 mg/d)	No rplC (ORF aa 84–217) mutation detected; no rrl (nt 2191–2929) mutation detected	2019 Nov 13	7.62 (18.25 h postdose)	24.15	17.88	12–26
Linezolid (600 mg MWF)	NT	2020 Mar 12	<2.00†	19.04	13.6	12–26

*MWF, Monday/Wednesday/Friday; NT, not tested; ORF, open reading frame; XDR, extensively drug-resistant tuberculosis. †Trough sample was not collected; based on the apparent elimination half-life, the linezolid concentration at 48 h was calculated to be <2 µg/mL, a value associated with minimal toxicity.

**Table 2:** Laboratory, electrocardiographic and clinical monitoring data for patient treated for XDR TB, United States*

Date	AST, IU/L	ALT, IU/L	ALP, IU/L	Creatinine, mg/dL	Magnesium, mg/dL	Hgb, g/dL	Leukocyte, × 10^9^/L	Plt, × 10^9^/L	QTc, msec	Notes
Initial treatment with rifampin, isoniazid, pyrazinamide, and ethambutol										
2019 Aug 2	67	86	221	0.66	NT	10.4	3.97	272	NT	None
Bridging regimen of bedaquiline, linezolid, moxifloxacin, cycloserine, clofazimine, and ethambutol										
2019 Oct 3	53	62	121	0.64	1.9	12.7	5.4	278	402	None
2019 Oct 15	73	70	147	NT	NT	12.6	5.3	243	NT	None
BPaL regimen										
2019 Oct 21	57	71	135	0.94	1.9	12.9	3.9	257	413	None
2019 Nov 11	40	53	113	1.01	1.9	12.3	4.9	237	426	Pantoprazole started for brief nausea
2019 Dec 2	52	63	118	0.77	1.9	10.7	4.3	289	421	None
2019 Dec 16	NT	NT	NT	NT	NT	11.2	4.7	267	NT	None
2020 Jan 6	58	74	107	0.80	2.0	12.5	5.1	271	422	None
2020 Feb 3	36	39	89	0.76	1.9	12.2	4.2	258	429	None

*Initial treatment period was August 5–September 9, 2019; bridging regimen period October 7–21, 2019; BPaL treatment period October 21, 2019–April 21, 2020. ALP, alkaline phosphatase; ALT, alanine aminotransferase; AST, aspartate aminotransferase; BPaL, bedaquiline, pretomanid, and linezolid; Hgb, hemoglobin; leukocyte, leukocyte count; NT, not tested; Plt, platelet; QTc, corrected QT interval.

The patient, physicians, and public health staff involved reported high satisfaction with BPaL. Providers and TB programs in the United States considering this regimen for TB patients can seek guidance from CDC Division of Tuberculosis Elimination or their TB COE. Current trials using BPaL, such as ZeNix (https://www.tballiance.org/portfolio/trial/11883), are evaluating lower doses and shorter duration of linezolid compared with those of the Nix-TB trial. The 6-month, all oral, highly effective BPaL regimen is a notable advancement toward reducing global TB deaths (*10*).
